# Psychiatric neurosurgery and the Theory of Mind network: ethics in the suffering, refractory patient

**DOI:** 10.1590/1980-5764-DN-2025-0465

**Published:** 2026-08-24

**Authors:** Julio César López-Valdés, Alfonso Arellano-Mata, Erik Rodrigo Velásquez-Coria, Daniel Alejandro Vega-Moreno, Laura Mestre-Orozco

**Affiliations:** 1 Universidad Nacional Autónoma de México, Faculty of Medicine, Postgraduate Department, Mexico City, Ciudad Universitaria, Mexico.; 2 Centro Médico Nacional Siglo XXI, Hospital de Especialidades “Dr. Bernardo Sepúlveda Gutiérrez”, Stereotaxic & Functional Neurosurgery Department. Cuauhtémoc, Mexico City, Mexico.; 3 University of Tamaulipas, Faculty of Medicine “Dr. Alberto Romo Caballero”, Research Department, Tampico, Tamaulipas, México.; 4 Universidad de la Salud, Postgraduate Department. Álvaro Obregón, Mexico City, Mexico.; 5 High Specialty Central South Hospital, PEMEX, Neurosurgery Department, Mexico City, Tlalpan, Mexico.; 6 Centro Médico, ISSEMYM, Oncological Neurosurgery Department, San Jerónimo Chicahualco, Mexico State, Mexico.; 7 The American British Cowdray Medical Center, Surgical Pathology Department, Cuajimalpa, Ciudad de México, México.

**Keywords:** Psychosurgery, Theory of Mind, Bioethics, Deep Brain Stimulation, Psicocirugía, Teoría de la Mente, Bioética, Estimulación Encefálica Profunda

## Abstract

Psychosurgery has evolved from imprecise lesional procedures to targeted stereotactic and neuromodulation interventions, with significant improvements in precision, safety, and ethical governance. Parallel advances in social neuroscience have mapped out the Theory of Mind (ToM) networks that support the attribution of mental states, empathy, and moral judgment. This article examines the historical progression of psychosurgery, links it to the neural correlates of ToM, and proposes a bioethical framework to mitigate risks to a patient’s agency and personal identity.

## RESUMEN.

La psicocirugía ha evolucionado desde procedimientos lesionales imprecisos hacia intervenciones estereotácticas y de neuromodulación dirigidas, con mejoras significativas en precisión, seguridad y gobernanza ética. Avances paralelos en la neurociencia social han mapeado las redes de la Teoría de la Mente (ToM) que sustentan la atribución de estados mentales, la empatía y el juicio moral. Este artículo examina la progresión histórica de la psicocirugía, la vincula con los correlatos neuronales de la ToM y propone un marco bioético para mitigar los riesgos para la agencia y la identidad personal del paciente.

## INTRODUCTION

 Today, functional neurosurgery has established itself as a valuable therapeutic option for managing various neurological and psychiatric diseases that severely compromise patients’ quality of life. Its application extends to conditions such as chronic pain, movement disorders (e.g., Parkinson’s disease), and severe neuropsychiatric conditions refractory to other treatments (e.g., major depression, obsessive-compulsive disorder, anorexia-bulimia nervosa). 

 This field experienced a notable resurgence in the treatment of psychiatric disorders, adopting a more precise and less invasive approach. Historically, the term “psychosurgery” was coined to define the use of neurosurgical procedures aimed at modifying brain circuits for therapeutic purposes in refractory psychiatric disorders^1^ . It is essential to note that this term was replaced by more precise and contemporary designations such as “psychiatric neurosurgery” or “neurosurgery for mental disorders,” reflecting an evolution in the understanding, techniques, and ethical standards of this specialty^1-4^. 

 The contemporary renaissance of neurosurgery for mental disorders (mainly for depression and obsessive-compulsive disorder) led to the rethinking of new treatments, ranging from minimal invasive approaches, such as transcranial magnetic stimulation, to lesional surgeries in specific deep brain regions; that is, psychosurgery moved from non-selective lesional procedures to stereotactic interventions and circuit neuromodulation, with substantial improvements in precision, safety, and ethical governance. This progress was largely possible thanks to the evolution of technology and a deeper understanding of the biology of mental illnesses^3- 5^. 

 In parallel, advances in social neuroscience outlined the neural networks that support the attribution of mental states, empathy, and moral judgment (Theory of Mind – ToM). However, this progress does not come without its own complexities. Neurosurgery for mental disorders poses important ethical challenges that require careful consideration. From Luigi Lanzarini (early 1800s)^6^ and his melancholy to the prefrontal leukotomies of the mid-20th century proposed by António Caetano de Abreu Freire Egas Moniz (1874–1955), there was a transition to high-precision approaches based on neuroimaging, stereotactic, and, increasingly, adjustable neuromodulation (e.g., deep brain stimulation – DBS)^3-8^ . 

 However, new ethical challenges emerged, such as informed consent, the establishment of the efficacy of these procedures from the literature and in the design of new studies, the relationship between harm and benefit, and the role of institutional and governmental regulatory control over psychosurgery. 

### Historical overview of psychosurgery

#### Early Era (1935–1960)

 Egas Moniz’s leukotomies and prefrontal lobotomy gained traction mainly due to the desperate shortage of effective therapeutic alternatives for severe psychiatric disorders at the time^7^ . The predominant medical ethic was often paternalistic, in which the doctor’s decision was paramount, and relief of patient suffering was sought even at the cost of significant side effects; however, the results of these interventions were notoriously inconsistent^9^ . 

 Even more concerning were the devastating side effects that tended to undermine patients’ autonomy and true decision-making capacity. Among these effects were abulia (lack of will or initiative), apathy (indifference), disinhibition (loss of control over social behavior), and executive deficits (problems with planning, organizing, and controlling behavior). These consequences often transformed individuals’ personality and functionality, leading to an eventual repudiation of these practices as pharmacological treatments emerged and more rigorous ethical standards were developed^1,10, 11^. 

 The lack of a deep understanding of brain circuits and the crudeness of the surgical techniques of the era contributed to the discredit of early psychosurgery. 

#### Decline and criticism (1960–1980)

 The era of the decline of psychosurgery, spanning approximately 1960 to 1980, was a crucial period in the history of this practice. After the initial enthusiasm and widespread diffusion of lobotomies in the early era, a series of factors converged to cause their discredit and a drastic reduction in their application^1-3^. 

 One of the most significant elements was the emergence and improvement of psychopharmacology. The development of more effective antipsychotic and antidepressant medications with fewer serious side effects offered a less invasive and more controllable therapeutic alternative for many psychiatric disorders that could previously only be treated with surgery. This drastically reduced the need to resort to brain surgical interventions^3-7^. In parallel, more detailed and critical reports on the functional damage that psychosurgery caused in patients began to accumulate. The consequences, such as abulia, apathy, disinhibition, and executive deficits, already mentioned in the early era, were documented more systematically. These reports highlighted how surgery, instead of “curing,” often left individuals with a reduced capacity to function autonomously and fully in society. 

 Finally, and perhaps the most determining factor, was the growing attention and awareness of human rights. As Western societies advanced in the protection of individual rights, the idea of permanently altering a patient’s brain without fully informed consent and with questionable results became unacceptable. Critical voices, including those of patients, family members, and human rights advocates, gained strength, leading to significant regulatory restrictions and a persistent stigma toward psychosurgery^5-7^. This stigma lasted for decades, marking the practice as an extreme and often dehumanizing measure. This period not only led to an almost complete halt in psychosurgery but also laid the foundations for a much more rigorous medical ethic, emphasizing patient autonomy, informed consent, and the need for solid evidence and clear benefits before any invasive intervention. 

 Nowadays, these negative historical backgrounds are a source of fear and anxiety for patients who are offered this treatment, which becomes a barrier to the doctor-patient relationship^12^ . To overcome it, it is essential for the doctor to offer an adequate explanation of the safety of current procedures, which are backed by rigorous evidence. 

#### Modern resurgence (1990–present)

 With the rise of stereotactic, functional magnetic resonance imaging (fMRI), and connectomics, more targeted procedures emerged: anterior cingulotomy, anterior capsulotomy (radiofrequency or radiosurgery), subcaudate tractotomy, amygdalotomy in exceptional cases, DBS in the ventral striatum/nucleus accumbens, internal capsule or cingulate, and non-invasive ablative techniques such as MR-guided focused ultrasound (MRgFUS)^11- 13^. 

 Current practice is governed by strict indications, consent protocols, and interdisciplinary committees. Lesions or dysfunctions in these areas can alter the understanding of intentions, sensitivity to social norms, and moral judgment. Fronto-striatal and limbic connectivity is crucial for translating mental inferences into motivation and behavior^2-4^. In addition, advances in neuroimaging allow a greater understanding of the brain correlates of mental disorders, which facilitate the identification of more precise targets for surgical intervention. 

 Although contemporary psychiatric neurosurgery has produced encouraging results in carefully selected patients — most notably in obsessive-compulsive disorder (OCD) and, to a lesser extent, treatment-resistant depression — the strength of the existing evidence remains uneven and insufficient to predict benefit at the individual level^14, 15^. Most clinical data come from small observational cohorts, heterogeneous protocols, and variable outcome definitions, which complicate the interpretation of therapeutic efficacy. Randomized controlled trials exist, particularly in OCD, but sample sizes remain modest and long-term follow-up is often incomplete, reinforcing the need for epistemic humility when discussing outcome expectations and ethical justification. Acknowledging these empirical limitations is essential for properly situating neurosurgical interventions within the broader therapeutic landscape of refractory psychiatric disease^14-17^. 

### Neurobiological bases of the theory of mind and its implications for brain function

 Advances in structural and functional connectomics have significantly improved the anatomical understanding of large-scale neural networks, yet their translational power for predicting complex psychological states or guiding psychiatric neurosurgery remains limited. While tractographic models reliably depict white-matter architecture, several methodological concerns persist, including inter-individual variability, low test–retest reliability, and the limited capacity of neuroimaging biomarkers to account for the multidimensional nature of mental illness. Consequently, connectomic precision cannot be equated with causal explanatory power, nor does network accuracy imply deterministic predictions about cognition, affect, or behavior. Integrating connectomic models with clinical phenomenology thus requires methodological caution and explicit recognition of their current epistemological limits^18-21^. 

 The Theory of Mind (ToM), understood as the ability to attribute mental states (beliefs, intentions, desires) to oneself and others to predict their behavior, is a fundamental pillar of social cognition^14^ . Although a comprehensive understanding of the functional correlation of ToM still presents challenges, especially concerning its intricate relationships with morality or empathy, current research has managed to elucidate a robust neural basis for the mentalization process^ 22,23^. 

**Figure 1.**
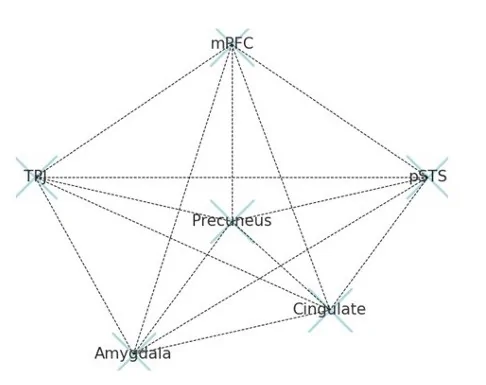
Connectivity of the main regions of the Theory of Mind network. The diagram shows the most relevant cortical and subcortical nodes: medial prefrontal cortex, temporoparietal junction, posterior superior temporal sulcus, precuneus, anterior cingulate, and amygdala. Dotted lines represent the functional interconnectivity between these areas, responsible for mental inference processes, empathy, intention attribution, and affective regulation in social interaction.

Abbreviations: mPFC, medial prefrontal cortex; TPJ, temporoparietal junction; pSTS, posterior superior temporal sulcus.

Source: Prepared by the authors based on the literature reviewed.

 Various studies reached a consensus on a set of specific brain regions that form the mentalization network. These include the right and left temporoparietal junction (RTPJ, LTPJ, respectively), the superior temporal sulcus (STS), especially the right (RSTS), the precuneus and the medial prefrontal cortex (mPFC). These areas work in concert to allow the understanding of the perspectives and internal states of other individuals (Figure 1)^22-24^. 

 In parallel, altered ToM is described in patients with neurodegenerative diseases. These conditions often significantly affect the orbitofrontal and cingulate frontostriatal circuits, as well as the mesolimbic dopaminergic system, which modulates the activity of these loops. This effect explains the dysfunction observed in the capacity for mentalization in these conditions^12,24 -26^. 

 On the other hand, studies of brain lesions provided strong evidence of the critical role of the frontal lobes in ToM skills. For example, damage to the orbitofrontal and/or ventromedial areas (OFC/vmPFC), both left and right, consistently causes profound personality changes that include indifference, significant impairment of social judgment, a marked decrease in the ability for emotional response, and deficient self-regulation. Specifically, the right frontal lobe is implicated in the appreciation of humor, self-awareness, facial self-recognition, and episodic memory. A paradigmatic example of this is the impairment of social cognition visible as a consequence of cerebrovascular disease, where the associated neurological changes disrupt the vital fronto-subcortical and fronto-temporal pathways that together make up the “social brain”, which often leads to disordered interpersonal functioning and poor regulation of personal behavior^27,28^. 

 Likewise, the vmPFC is strongly implicated in processes related to “moral judgment.” For this reason, it is attributed a crucial role in each subject’s personal attachment to social norms and cultural values, serving as an integrating center for socio-emotional information for ethical decision-making^22^ . 

### Dissociation of the cognitive and affective components of Theory of Mind

 Aside from the above, it is widely accepted that ToM is a dissociated process, composed of cognitive and affective elements that are distinguishable at a behavioral level and are presumably mediated by separate neural networks. Abu-Akel and Shamay-Tsoory^27^ proposes a more granular division, attributing specific areas for each function within the ToM. According to his model, there are brain regions involved only in the representation of one’s own mental states (TPJ, including inferior parietal lobe/parietal operculum), specific brain zones for the representation of others’ mental states (STS), and brain areas that are common for both tasks (amygdala, anterior cingulate gyrus, OFC, dorsomedial prefrontal cortex (dmPFC), vmPFC, and inferolateral frontal cortex). 

 From a functional point of view, neuroimaging studies provided convincing evidence of the breakdown between both components of ToM. These studies evaluated the processing areas of cognitive ToM versus affective ToM by examining their brain activity in response to specific tasks. For example, “false belief tasks” are used to evaluate cognitive ToM (the ability to infer what another believes, even if incorrect), and “*faux pas tasks*” for affective ToM (the ability to recognize a socially awkward situation or a lack of tact that involves the emotion of another)^23^ . 

 In this sense, Abu-Akel and Shamay-Tsoory^27^ point to evidence that the affective processing of ToM is located and developed mainly in the PFC, the OFC (Brodmann area 11/12/47) and the vmPFC (Brodmann area 10/32). Likewise, the dmPFC (Brodmann area 8/9), and the dorsolateral prefrontal cortex (dlPFC) (Brodmann area 9/46) are involved, mostly, as the processing areas of cognitive ToM^29,30^. In addition, the existence of dense connections between the amygdala and the participation of the vmPFC, the OFC, and the inferolateral frontal cortex suggests a strong involvement of the latter in the affective processing of ToM^31^ . 

### The role of the ventrolateral prefrontal cortex and competition with the dorsolateral prefrontal cortex

 Within the growing literature, various studies suggest that the ventrolateral prefrontal cortex (vlPFC) plays an important role in both executive and social functions. Mar^32^ even mentions it as a possible “core of the mentalization network,” particularly the left inferior frontal gyrus (IFG). It is postulated that this area has an influence on the understanding and selection of viewpoints belonging to both oneself and other people. That is, it mediates the integration of someone’s perspective of oneself with self-perception. In a simpler way, it helps in the creation of new criteria from preformed ones, accepting the inclusion of details from other people. Given this quality, as well as its ability to mediate moral criteria, it allows a response to be represented according to the perceived preferences and intentions of another subject. According to Prehn et al.,^33^ the left vlPFC is activated with greater intensity in subjects with a lower ability to apply moral principles in decision-making. 

 In contrast, the dlPFC, often referred to as the “rational area,” competes directly with the vmPFC to suppress the predominant emotional functions of the PFC in certain situations. This allows for a more objective analysis of each of the situations raised. In other words, the dlPFC is crucially involved in problem-solving, cognitive control of facts, cost-benefit analysis of decisions, and the evaluation of situations that involve rule-based knowledge and dishonest behavior^34^ . This is how Greene et al.^35,36^ and Prehn et al.^33^ mention that this area reflects the process of lying and the determination of deceptive responses, as well as the responsibility for crimes and other situations related to false facts. 

**Figure 2.**
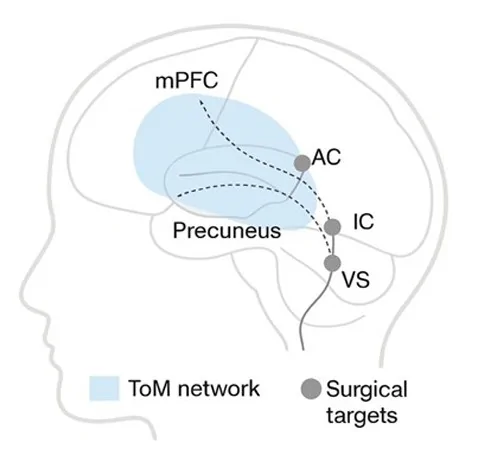
Theory of Mind networks and their overlap with neurosurgical targets. The main ToM circuit — medial prefrontal cortex and precuneus — is represented in blue, while the gray circles indicate functional surgical targets commonly used in the treatment of refractory psychiatric disorders: anterior cingulate, internal capsule, and ventral striatum/nucleus accumbens. The dashed lines illustrate the connections between both systems, underlining the anatomical and functional proximity that poses risks and opportunities in contemporary psychosurgery.

Abbreviations: ToM, Theory of Mind; mPFC, medial prefrontal cortex; AC, anterior cingulate; IC, internal capsule; VS/NAc, ventral striatum/nucleus accumbens.

Source: Prepared by the authors based on anatomical-functional correlation analysis.

 In summary, ToM is a multidimensional construct with a complex neural architecture. The understanding of its cognitive and affective components, and the identification of the underlying brain networks, are not only fundamental to the comprehension of normal social cognition, but also to unraveling the bases of the alterations observed in various neuropsychiatric and neurological diseases. Figure 2 shows the ToM network and its overlap with neurosurgical targets. 

### Intersections between psychosurgical targets and theory of mind networks

 ToM is the cornerstone of social life, empathy, and moral reasoning. Its neural networks partially overlap with surgical targets used in major depression, OCD, refractory pain, pathological aggression, or OCD with tics29. Examining this overlap is essential to prevent collateral effects that might impact agency and identity. This is particularly important because social life, empathy, and moral reasoning are the foundations of the patient’s narrative continuity. 

### Correlation with psychiatric neurosurgery

 The deep understanding of the complex neural network underlying the ToM and its cognitive and affective components is of critical relevance in the field of psychiatric neurosurgery. Given the ability of these interventions to modulate specific brain circuits such as the mPFC, OFC, the anterior cingulate gyrus, or the nucleus accumbens (NAc), all of which are involved in ToM, morality, and social cognition, it is imperative to consider the potential impact of surgery on these higher functions^37,38^. 

 Post-surgical alterations in personality traits, empathy, social judgment, or self-regulation, which were historically reported with lesional procedures and continue to be a concern with more modern techniques such as DBS or focal lesions (MRgFUS/laser interstitial thermal therapy – LITT), can be explained by the inadvertent or intentional modulation of these mentalization networks. Therefore, a detailed knowledge of the functional anatomy of ToM is not only crucial for identifying more precise therapeutic targets in psychiatric disorders with prominent social deficits (such as OCD or severe depression), but also for preventing or mitigating side effects that could compromise the patient’s authenticity, narrative continuity, and agency, thus ensuring an ethical and holistic surgical approach^35 -38^. 

 According to Abu-Akel and Bailey^30^ , clear separation is needed when discussing targets for Parkinson’s disease versus psychiatric conditions. The subthalamic nucleus (STN), globus pallidus internus (GPi), and ventral intermediate nucleus (Vim) are well-validated motor targets with extensive Level I and II evidence supporting their use. In contrast, targets such as the ventral capsule/ventral striatum (VC/VS), the NAc, or the subcallosal cingulate have been explored primarily for psychiatric disorders under different conceptual frameworks. Distinguishing these therapeutic contexts enhances the scientific clarity of the manuscript and avoids conflating anatomically adjacent but functionally distinct targets.^14,37 ,38^

### Contemporary clinical evidence

 When analyzing each of the implications that the ToM has within the various neuropsychiatric diseases, a multitude of executive functions and both social and functional mental processes with some well-known and described affectation can be perceived; however, within this brief summary, only those characteristic and determinant tergiversations of each of the entities of greatest interest in the current medical field will be addressed, this due to the limited space for this review (Table 1).^29,39^

**Table 1.** Overlap between neural modulations and effects on Theory of Mind (cognitive-social).

Technique	Modulated brain circuit/targets	Potential therapeutic effects	Side effects/theoretical risks on Theory of Mind and social functions
Anterior cingulotomy	Cingulate-subcortical circuit involved conflict, social pain, and emotional regulation	Reduction of anxious/obsessive hyperresponsiveness	Changes in error evaluation or painful empathy
Anterior capsulotomy	Fronto-thalamic fibers connecting the orbitofrontal cortex (OFC)/ medial prefrontal cortex (mPFC) with the thalamus and striatum	Impact on social evaluation, habit and compulsive control	Not directly specified, but implicit in the modulation of social evaluation and control
Deep brain stimulation of the nucleus accumbens/ventral striatum	Involved in reward, motivation and social learning	Influence on reward, motivation and social learning processes	Transient hypomania or changes in initiative/apathy, with potential impact on social sensitivity
Deep brain stimulation/capsulotomy in internal capsule (ventral)	Modulates the transfer of information between the prefrontal cortex and the basal ganglia	May alter salience processing, threat biases and cognitive perseveration	Not directly specified
Magnetic resonance-guided focused ultrasound/Laser Interstitial Thermal Therapy for select limbic targets	Focal lesions in select limbic targets, with tractography-guided planning	Allow precise lesions with advanced planning	The challenge of predicting effects on emerging social functions

Source: Prepared by the authors based on systematic literature data synthesis.

### Refractory obsessive-compulsive disorder

 OCD is one of the psychiatric disorders with the greatest functional impact and one of those that most motivate the development of neurosurgical interventions when conventional treatments fail (intensive cognitive-behavioral psychotherapy and at least three pharmacological regimens with selective serotonin reuptake inhibitors, clomipramine, and potentiators)^40^ . 

 In recent years, various studies showed that OCD is not limited to dysfunctions in the fronto-striatal and orbitofrontal circuits, but also involves alterations in social cognition and, in particular, in ToM; they showed difficulties in tasks of inferring mental states, especially in recognizing intentions and interpreting ambiguous social signals^41^ . These alterations are related to the hyperactivity of the OFC, the anterior cingulate, and the basal ganglia, but also to a relative hypoactivation of classic ToM networks, such as the TPJ and the mPFC^41 ,42^. This would explain the tendency toward rigid hypermorality and hyper-responsibility characteristic of some OCD subtypes, where the difficulty is not a lack of empathy, but an overestimation of guilt and moral duty. 

 In addition, alterations in ToM can contribute to the persistence of compulsive rituals as maladaptive strategies to reduce anxiety in the face of uncertainty about the intentions or judgment of others. In therapeutic settings, these difficulties can limit the therapeutic alliance and the effectiveness of cognitive interventions, as the patient tends to rigidly interpret the perspective of others^24,39,43 -46^. 

 In the particular case of OCD, neurosurgical interventions (cingulotomy, capsulotomy, DBS) impact regions that participate in both affective regulation and affective ToM (especially anterior cingulate and limbic connections). Bioethically, this link requires the strengthening of informed consent, warning that surgery can not only modify obsessive-compulsive symptoms, but also aspects of social interaction and the patient’s moral self-perception^24,40 ,43-45^ . 

### Resistant major depression

 There is well-established evidence that indicates structural and functional alterations in the brain networks in depression that include abnormalities in the vmPFC and temporoparietal junction (TPJ)^47^ . Within the functions of ToM, the available evidence suggests that the deficit is not limited to affective or cognitive stimuli, but the perception of stimuli for the coding of ToM reasoning is directly modified, which is why social inabilities exist in both verbal and visual tasks (e.g., reading faces). Likewise, Bora and Berk^47^ , indicate that said modifications persist after the remission of the depressive event, which can be a risk factor for subsequent relapses. DBS directed at the subcallosal cingulate (area 25), ventral internal capsule, and ventral striatum produces variable responses; current protocols emphasize individual connectivity selection, gradual programming, and patient-centered outcome measurement^1,3 ,47^. 

### Parkinson’s disease

 It is well known that, within Parkinson’s disease, there is an evident neurological deterioration that includes both motor and cognitive areas. However, the different deficiencies present in a large number of social skills are little known, such as emotional prosody, the deficit in the recognition of emotions through the facial expression of others, the understanding of irony and sarcasm, as well as certain activities that involve decision-making in social scenarios. In a certain way, the patient who suffers from the condition is “socially blind,” since he is unable to generate a response in accordance with the emotional stimulus perceived, due to his “limited” empathy as a result of the inability to read and generate facial expressions. Therefore, the patient with Parkinson’s disease has a limited work function not only because of the motor and cognitive conditions, but also because of the social restrictions that their illness entails^48-50^. 

 The surgical treatment of these patients presents a clear ethical dilemma. Despite the existence of Level I evidence supporting the efficacy of bilateral DBS in the STN, and Level II evidence for implantation in the NAc, a critical gap in scientific knowledge remains^51 ,52^. 

 It is essential to distinguish that while the STN is a validated motor target, its application in psychiatric contexts often requires specific coordinates, such as 2 mm anterior and 1 mm medial to the traditional Parkinsonian target. This distinction is critical because the STN and the NAc involve different functional frameworks and stereotactic targets, and conflating them may lead to a misunderstanding of the intended therapeutic modulation. 

 Although these techniques are safe and validated by literature, there is no evidence to guide the selection of which patients would benefit from each. Databases and published studies to date do not include direct comparisons between the two techniques, making it impossible to precisely determine the most suitable surgical approach for each patient profile. This lack of information places the surgeon in a position where a treatment with a considerable level of evidence for its safety and efficacy can be offered, yet without the necessary scientific support for an informed decision regarding the choice of surgical target. Consequently, this scenario underscores the urgent need for prospective, randomized studies to compare both techniques in order to establish clear patient selection guidelines. 

### Pathological aggression

 Pathological aggression constitutes one of the most disruptive symptoms in a small subgroup of patients with refractory epilepsy, severe neuropsychiatric disorders, or acquired brain damage. It is characterized by recurrent, disproportionate, and unmanageable violent behaviors not controlled by conventional strategies (pharmacotherapy, psychotherapy, behavioral measures, or physical containment). 

 In these cases, the risk of harm to third parties and the inability to maintain an autonomous life justify the consideration of exceptional neurosurgical interventions. In exceptional cases, amygdalotomy or limbic targets have been used with reinforced ethical scrutiny. The priority is to exhaust alternatives and establish a functional prognosis, the risks of disinhibition, and a plan of safeguards^31-36,53^. 

 Pathological aggression lies in a borderline area of psychosurgery; it can protect the patient and others by reducing uncontrollable violence. However, it also poses the dilemma of surgically intervening in emotional identity, with the risk of altering the subject’s authenticity^54^ . The balance between benefit and potential harm is key. Therefore, international organizations and ethics committees recommend reserving these interventions for extraordinary, documented, and supervised cases, more as a last resort than as standard clinical practice^54-56^. 

### The missing dimension: the patient with refractory suffering

 Severe, treatment-refractory psychiatric illness profoundly disrupts agency, motivation, affective experience, and interpersonal functioning long before surgical intervention is considered. Far from being ethically “neutral,” the illness itself erodes core attributes such as autonomy, self-coherence, moral agency, and social cognition — features often emphasized in the ethical critique of psychosurgery. Evidence shows that chronic, multi-resistant depression, OCD, and other severe disorders are associated with high suicide risk, marked functional disability, and neurocognitive distortions that compromise decision-making far more than the potential side effects of neurosurgical intervention. A robust ethical analysis must therefore take into account not only the risks of altering identity through neuromodulation but also the harms of withholding established therapies from patients whose suffering is prolonged, debilitating, and sometimes lethal. 

 In cases of extreme and refractory psychiatric illness, where multiple evidence-based therapies have failed, the clinical and ethical calculus shifts. Here, the principle of *primum non nocere* must be weighed against the moral imperative to alleviate unremitting suffering. For some patients, the alternative to neurosurgical intervention is persistent incapacitating disease or suicide. In such contexts, offering a well-established and comparatively safe neurosurgical procedure may constitute a compassionate and ethically justified course of action (*melius anceps remedium quam nullum*), provided that rigorous selection, informed consent, and multidisciplinary evaluation are maintained. 

### Bioethical framework: from principles to the person

#### Classic principles of bioethics and their expansion in neurosurgical contexts

 Today, bioethics is a discipline of exponential growth, emerging as a fundamental area within medicine, particularly in the demanding environment of the hospital clinic. Here, a deep understanding of the basic concepts of the human “being” and the need for ethical and reflexive decision-making become imperative. Every day, routine clinical decisions require meticulous attention to ensure the most favorable scenario possible for the patient, respecting their dignity and rights^57, 58^. 

 Bioethics and medical philosophy provide essential conceptual frameworks that serve as a compass to guide clinical practice, ensuring that respect for autonomy, dignity, and equity are unwavering pillars^58, 59^. The four classic principles of bioethics (beneficence, non-maleficence, autonomy, and justice), established by Beauchamp and Childress^60^ , remain valid and constitute the basis of ethical deliberation in medicine. The bioethical framework must be expanded to include the evolving field of neuroethics and neurorights^61^ . This perspective demands explicit protection of cognitive liberty, mental integrity, and psychological continuity, particularly when interventions modulate circuits underlying self-governance. By integrating these neuroethical constructs, the clinician can better balance the protection of a patient’s personal identity with the moral imperative to alleviate the profound suffering caused by refractory illness. 

 However, when applied to fields as complex and delicate as psychiatric neurosurgery, these principles demand a nuanced interpretation and, on occasion, an expansion. 

 Psychiatric neurosurgery, due to its inherent ability to alter fundamental traits of an individual’s personality, motivation, and practical morality, poses ethical dilemmas that go beyond the direct application of classic principles. 

 Recent discussions on neurorights provide an essential contemporary framework for interpreting the ethical challenges of psychiatric neurosurgery. In their 2025 analysis, *Neuroethics and Neurorights* ^61^ , the authors argue that advances in neurotechnology demand explicit protection of cognitive liberty, mental integrity, psychological continuity, and personal identity, particularly when interventions have the capacity to modulate neural circuits underlying agency and self-related processes. These concerns are directly relevant to psychiatric neurosurgery, where targets frequently intersect with networks implicated in ToM, affect regulation, and decision-making. From this perspective, ethical evaluation must extend beyond procedural safety to include safeguards against unintended alterations of mental autonomy and narrative identity, while simultaneously recognizing that severe, refractory psychiatric illness itself may already violate these neurorights through persistent suffering, loss of self-governance, and diminished social cognition. Integrating a neurorights-based framework therefore strengthens the ethical justification for neurosurgical intervention by balancing protection of mental integrity with the moral obligation to alleviate otherwise intractable suffering. 

 The possibility of modifying the essence of who a person is forces the incorporation of additional ethical considerations, such as authenticity, “narrative continuity,” and agency^60-64^. All of this is because, beyond the autonomy to decide, the question arises whether the surgical intervention could compromise the authenticity of the patient’s being, that is, their true “self.” Does the person who emerges after surgery remain fundamentally the same, or were they transformed in a way that affects their sense of identity and purpose? This is why the concept of authenticity is vital to evaluate whether the therapeutic “benefit” does not entail an irreversible loss of the patient’s “self”^60-65^. 

 Furthermore, it is important to remember that human identity is often built through a narrative continuity of experiences, memories, and future plans. A significant alteration of personality or memory due to psychosurgery could fragment this narrative, affecting how the patient perceives themselves over time. Thus, it must be considered fundamental how the intervention could impact the coherence of the individual’s life story. 

 It is essential to contextualize that devastating effects such as apathy, abulia, impaired decision-making capacity, and social withdrawal are intrinsic to advanced psychiatric disorders and not solely consequences of neurosurgical intervention. Severe OCD, depression, and related conditions can profoundly erode autonomy and volition, often leaving patients functionally incapacitated. Any ethical analysis that locates threats to agency exclusively in the surgical procedure risks overlooking the substantial harms inflicted by the disease itself^66,67^. 

 On the other hand, agency refers to an individual’s ability to act independently and make decisions based on their own intentions and values. If psychosurgery modifies motivation or the capacity for judgment, it could reduce the ability to exercise control over one’s own life and choices. This raises questions about the patient’s true freedom in the post-surgical decision-making process. 

 These expanded principles provide an essential and sophisticated guide for ethical decision-making in psychiatric neurosurgery, especially in situations in which the values and preferences of patients, medical objectives, and the profound implications of the intervention may be in conflict. Bioethical deliberation in this field not only seeks to protect the patient from harm but also to preserve their humanity, their agency, and their sense of self within the complex intertwining of mind and brain. The need for informed consent that encompasses these profound dimensions is, therefore, more critical than ever. 

### The multidisciplinary committee and decision-making

 To ensure the appropriate selection of patients for invasive procedures, a multidisciplinary committee must make the decisions. This team should include neurologists, neuropsychiatrists, psychiatrists, clinical psychologists, and health professionals with training in bioethics. Although crucial to the committee, these professionals do not have daily clinical experience with irreversible procedures that can have a high impact on a patient’s life, unlike the functional neurosurgeon^68^ . 

 This team must establish a shared and comprehensive source of knowledge regarding the neurophysiological and neuroanatomical basis of each procedure. This must be individualized to the patient’s comorbidities, family, social, and socioeconomic context, with well-defined goals when making a final recommendation. This recommendation must never attempt to override the patient’s principle of autonomy^68 ,69^. 

### Ethical dilemmas in research and clinical practice

 An important bioethical aspect is offering a safe, invasive procedure to patients with the aforementioned histories, supported by robust scientific evidence to adhere to the principles of beneficence and non-maleficence. However, obtaining this level of evidence entails certain difficulties in the field of psychosurgery. 

 The ethical debate surrounding psychosurgery must explicitly acknowledge that the integrity of patients undergoing these procedures is often already profoundly compromised by the severity of their illness. Disorders such as refractory OCD or major depression can diminish motivation, impair moral reasoning, distort self-perception, and weaken social cognition. Thus, the risk of altering identity through surgery must be weighed against the reality that these aspects of identity are already eroded by the disease itself. Presenting psychosurgical interventions as threats to otherwise intact individuals generates a misleading ethical narrative that does not reflect the lived reality of clinical practice^14,68^. 

 Including patients in randomized clinical trials presents an ethical dilemma, as it involves subjecting patients without a focal neurological deficit to a surgical procedure deep within their brain, which could result in a postoperative deficit. Furthermore, the safety of clinical studies cannot be fully substantiated by prior basic science research, as animal models do not adequately represent most of the social and intellectual functions affected in neuropsychiatric disorders. Finally, these are innovative procedures that require including a larger patient population to achieve a higher level of scientific evidence^70,71^. 

 An ethically balanced perspective involves examining not only the possible harms of performing psychosurgery but also the harms of denying it. Failure to refer severely ill, treatment-refractory patients to established neurosurgical options — such as capsulotomy, cingulotomy, or DBS — may prolong suffering, exacerbate disability, and in some cases contribute to suicide risk. Ethical responsibility therefore includes ensuring access to treatment, combating stigma, and avoiding paternalistic withholding of potentially beneficial interventions. Leksell’s classic argument, “asserting that ideology should not prevent a patient from receiving empirically beneficial care”, remains relevant in modern psychosurgery^72^ . 

 Although informed consent is not a novel ethical challenge, it takes on particular complexity in psychiatric neurosurgery, in which the patient’s decisional capacity may fluctuate and the interventions may carry potential implications for identity, affect, and agency. Contemporary guidelines emphasize the importance of capacity assessment, iterative consent processes, involvement of caregivers, and clear disclosure of the limits of current evidence. Presenting informed consent as a renewed challenge rather than a new one more accurately reflects its enduring centrality in the field^70-72^ . 

### Economic barriers and access

 The economic context is a factor to consider in psychosurgery decision-making, representing a significant barrier to accessing this technology in low- and middle-income countries. On one hand, there is a lack of neurosurgeons in these regions, with a density of 0.120.37 neurosurgeons per 100,000 inhabitants^67^ . The density of functional neurosurgeons is even lower, to the extent that some countries have no medical personnel trained in this field. Training in these geographical areas often requires migration to high-income countries, with the added difficulty of securing economic resources for self-funded training. Additionally, the high cost of the necessary psychosurgery devices is a major challenge; in most cases, access is only possible through institutional support or medical insurance coverage. To mitigate this difficulty, strict adherence to international bioethical recommendations could allow for the inclusion of the manufacturing industry for research purposes, avoiding conflicts of interest. 

### The reductionism of the mind

 The pathophysiology of psychiatric diseases involves a multifactorial basis with an interaction between biological, genetic, and psychosocial origins. One of the limitations of neurosurgical treatment is the reductionism of the mind to a biological substrate. Neuroscientists consider it ethically correct to offer psychosurgery to appropriately selected patients to provide a therapeutic alternative with a possibility of improvement, specifically for patients who have no other therapeutic options and for whom the procedures have been shown to be safe^14,22,70^. Due to the immense complexity of the mind, psychosocial function, and the concept of the “self,” as in all ethical debates, it may never be possible to adequately answer what is right and what is not. However, it is necessary to continue to pose questions and foster research to improve decision-making in this field. 

 Interpreting connectomic models as deterministic frameworks that predetermine human behavior risks oversimplifying the multilevel architecture of agency. Contemporary neuroscience suggests that while neural networks shape cognitive dispositions and constrain possible actions, they do not fully account for the dynamic interplay between biological substrates, developmental trajectories, environmental context, and deliberative capacities. Ethical analyses therefore increasingly favor compatibilist perspectives, acknowledging biological influences without reducing agency to a mechanistic epiphenomenon. This approach accommodates neuroscientific findings while preserving the normative foundations of autonomy, responsibility, and informed consent that underlie modern psychiatric practice^72-75^. 

 In conclusion, contemporary psychosurgery, supported by stereotactic precision, advanced neuroimaging, and adjustable neuromodulation, represents a therapeutic option of great value for patients with refractory psychiatric and neuropsychiatric disorders. In the face of intense suffering and marked functional disability, these interventions can open a window of opportunity when other alternatives have failed. However, their anatomical and functional proximity to the circuits that sustain the ToM, social cognition, and moral judgment poses a unique challenge. The possibility of inadvertently modifying traits that support an individual’s authenticity, agency, and narrative continuity requires a more careful approach than in other areas of functional neurosurgery. 

 In short, the ultimate goal of contemporary psychosurgery should not be limited to the reduction of refractory symptoms but should aspire to preserve the dignity, identity, and agency of the patient within their moral biography. The challenge is not only technical but profoundly human: to offer relief without compromising what constitutes the very core of the person. 

 The future of psychiatric neurosurgery lies in transitioning from a purely symptom-centered model to one that prioritizes the restoration of “relational authenticity” and the protection of neurorights. As our understanding of the overlap between ToM networks and surgical targets matures, clinical protocols should incorporate specific social-cognitive assessments, such as empathy and moral judgment batteries, to monitor the functional integrity of the “social brain” postoperatively. Furthermore, addressing the global imbalance in access to these technologies is not merely a technical challenge but a bioethical imperative of distributive justice, particularly in regions where the density of functional neurosurgeons remains critically low. Ultimately, by acknowledging that severe refractory illness itself, rather than the intervention, is often the primary constraint on a patient’s agency, neurosurgery can be recontextualized as a tool for reclaiming the patient’s narrative continuity and moral biography. 

## Data Availability

The datasets generated and/or analyzed during the current study are available from the corresponding author upon reasonable request.
